# Prenatal diagnosis and perinatal outcomes of umbilical cord hemangioma: two case reports and a literature-based perspective

**DOI:** 10.3389/fmed.2026.1847713

**Published:** 2026-07-08

**Authors:** Xing Xu, Hongbei Wan, Rong Tian, Xiaohong Lu, Hongxia Yuan

**Affiliations:** Department of Ultrasound, Changsha Hospital for Maternal and Child Health Care Affiliated to Hunan Normal University, Changsha, China

**Keywords:** Doppler imaging, fetal outcome, intrauterine fetal death, prenatal ultrasound, umbilical cord hemangioma, vascular architecture

## Abstract

Umbilical cord hemangioma is a rare benign vascular tumor associated with potentially serious perinatal complications; however, the prognostic significance of specific imaging findings and the optimal strategies for prenatal surveillance and perinatal management remain incompletely defined. We report two cases of umbilical cord hemangioma diagnosed prenatally by ultrasonography and confirmed by postnatal histopathological examination. In both cases, the lesion presented as fusiform enlargement of the umbilical cord with mixed echogenicity, comprising hyperechoic nodular components and cystic changes. Advanced Doppler imaging demonstrated branching intralesional vascular channels, facilitating recognition of the lesion. Despite apparently reassuring conventional Doppler indices in one case, intrauterine fetal demise occurred at 36 weeks, most likely related to progressive vascular compression. In the second case, right umbilical artery thrombosis and progressive cystic enlargement were detected on serial examinations, and a live infant was delivered at 33 weeks. These findings suggest that a recognizable sonographic pattern, together with detailed vascular assessment, may aid in the prenatal identification of umbilical cord hemangioma. Importantly, our observations suggest that conventional Doppler parameters alone may not fully reflect evolving hemodynamic compromise in some cases. Close follow-up with attention to structural evolution and signs of vascular compromise may assist clinical management, although validated prognostic markers are cuCrrently lacking.

## Introduction

Umbilical cord hemangioma is an exceptionally rare benign vascular tumor arising from the endothelial cells of umbilical vessels and is frequently associated with edema of Wharton’s jelly (1). Histologically, it is characterized by the proliferation of vascular channels within the umbilical cord stroma (1, 2). Although characteristic prenatal sonographic features have been described in previous reports, the prognostic significance of specific imaging findings and the optimal strategies for prenatal surveillance and perinatal management remain incompletely defined (3). Here, we report two cases of umbilical cord hemangioma diagnosed prenatally by ultrasonography and confirmed by postnatal histopathological examination. We summarize their sonographic characteristics and pregnancy outcomes and discuss the diagnostic challenges, prenatal surveillance strategies, and clinical management considerations in the context of the existing literature.

## Case 1

Ultrasound examinations were performed using a Voluson E10 system (GE Healthcare) equipped with C1-5-D (1–5 MHz) and C2-9-D (2–9 MHz) convex transducers, as well as an RM6C volume transducer (2–8 MHz).

**FIGURE 1:**
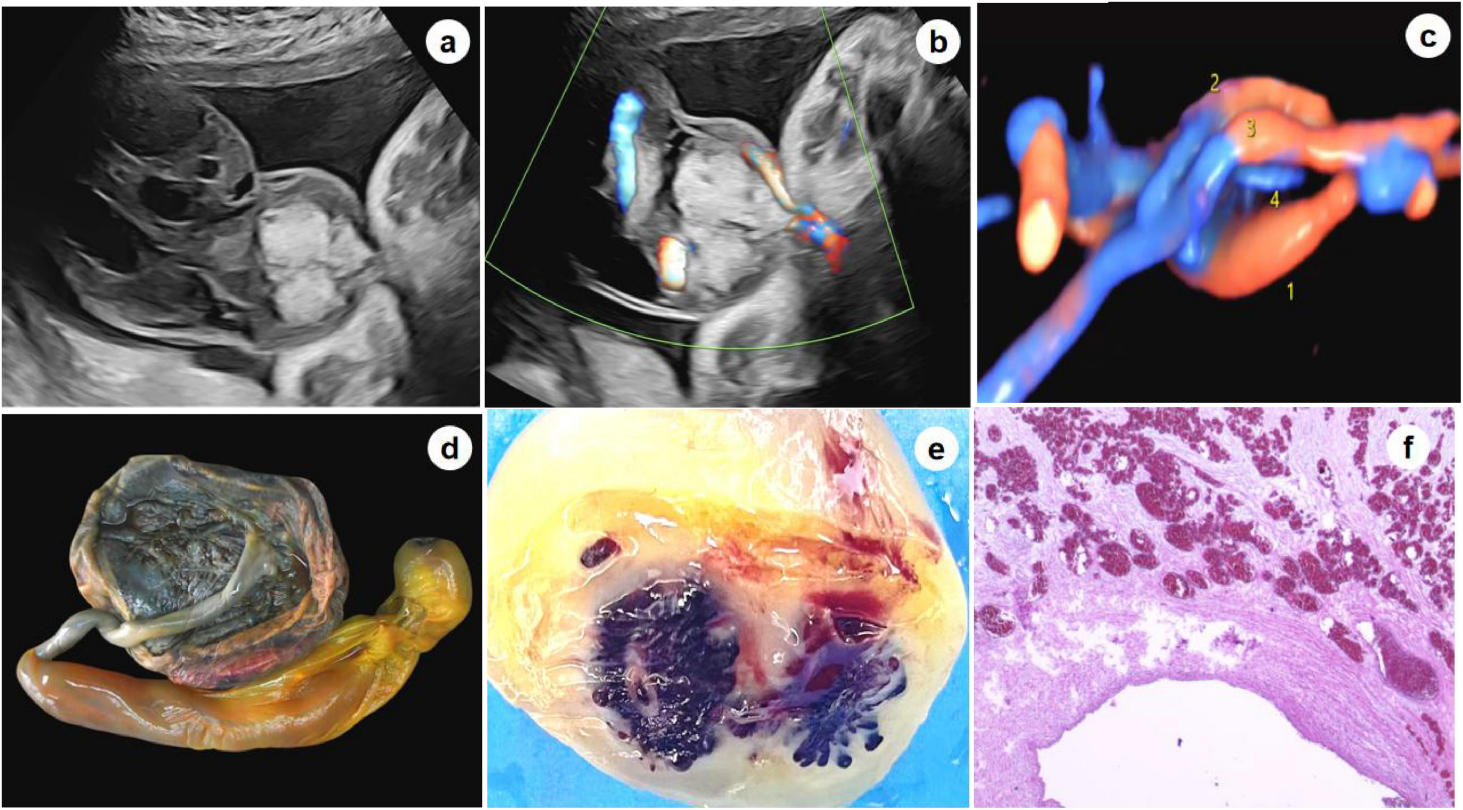
Prenatal ultrasonographic, gross, and histopathological findings of umbilical cord hemangioma in Case 1. Two-dimensional ultrasonography **(a)** showing fusiform enlargement of the umbilical cord near the fetal abdominal insertion, with thickened Wharton’s jelly and internal hyperechoic nodules. Color Doppler imaging **(b)** showing the umbilical vessels traversing the lesion. STIC-HDlive Flow imaging **(c)** demonstrating four prominent vascular channels within the affected segment. Gross examination at delivery **(d)** demonstrating the affected segment near the fetal insertion and the uninvolved segment near the placental end; cross-section **(e)** showing nodular lesions compressing the umbilical vessels. H&E section **(f)** showing proliferating vascular channels embedded within edematous Wharton’s jelly, consistent with umbilical cord hemangioma.

A 27-year-old primigravida underwent routine prenatal anomaly screening at 23 weeks of gestation. Fetal anatomy was unremarkable, and biometric parameters were appropriate for gestational age. Ultrasonography revealed a fusiform enlargement of the umbilical cord located 1.3 cm from the fetal abdominal insertion (shown in Figure 1a), measuring 7 cm in length and 2.8 cm in diameter. The Wharton’s jelly appeared thickened, containing two hyperechoic nodules measuring approximately 1.0 × 0.6 and 1.2 × 0.7 cm. While color Doppler detected low-velocity flow within the nodules, spectral Doppler evaluation demonstrated normal waveforms in the umbilical arteries, middle cerebral artery, and ductus venosus.

Serial ultrasound examinations were performed every 2 weeks. At 31 weeks, the lesion had markedly enlarged, measuring approximately 24 cm in length with a maximum diameter of 6.3 cm. Wharton’s jelly appeared edematous and thickened. Three hyperechoic nodules were noted, measuring approximately 2.9 × 1.3, 2.0 × 1.2, and 3.1 × 1.3 cm, which surrounded the umbilical vessels that traversed the lesion (shown in Figure 1b). In the unaffected segment, two umbilical arteries and one umbilical vein were identified, whereas the affected segment showed four conspicuous vascular channels, suggestive of the presence of relatively large neovascular channels. Spatiotemporal Image Correlation with High-Definition live Flow (STIC-HDlive Flow) imaging clearly delineated the three-dimensional course and spatial relationships (shown in Figure 1c). Concurrently, spectral Doppler waveforms of both umbilical arteries, the middle cerebral artery, and the ductus venosus remained within normal limits, and fetal biometry was consistent with gestational age. Given the marked interval enlargement of the lesion and progressive edema of Wharton’s jelly, surveillance was intensified to weekly ultrasound examinations. Fetal biometry and Doppler indices remained stable, with no further lesion progression until 35 weeks of gestation. At 36 weeks, however, ultrasonography revealed intrauterine fetal demise, prompting labor induction. Following delivery, a mass was observed near the fetal insertion of the umbilical cord (shown in Figure 1d). Pathological examination revealed a normal umbilical architecture with two arteries and one vein (2A1V). The distal segment appeared markedly edematous and enlarged, with nodular lesions evident on the cut surface, causing compression of the umbilical vessels (shown in Figure 1e). Microscopic examination confirmed the diagnosis of an umbilical cord hemangioma, characterized by densely proliferating vascular channels forming an anastomosing network within edematous Wharton’s jelly (shown in Figure 1f). The placenta weighed 390 g (25th–50th percentile for gestational age) and showed delayed villous maturation. Placental histopathological examination revealed reduced villous vascular perfusion, focal avascular villi, and increased nucleated fetal erythrocytes, findings suggestive of chronic fetal circulatory compromise. The fetal weight was 2,250 g (fetal-to-placental weight ratio, 5.8), and the total umbilical cord length was approximately 40 cm. No fetal autopsy was performed; pathological evaluation was limited to the placenta and umbilical cord. External examination revealed no cutaneous hemangiomas or other clinically apparent vascular malformations.

## Case 2

**FIGURE 2:**
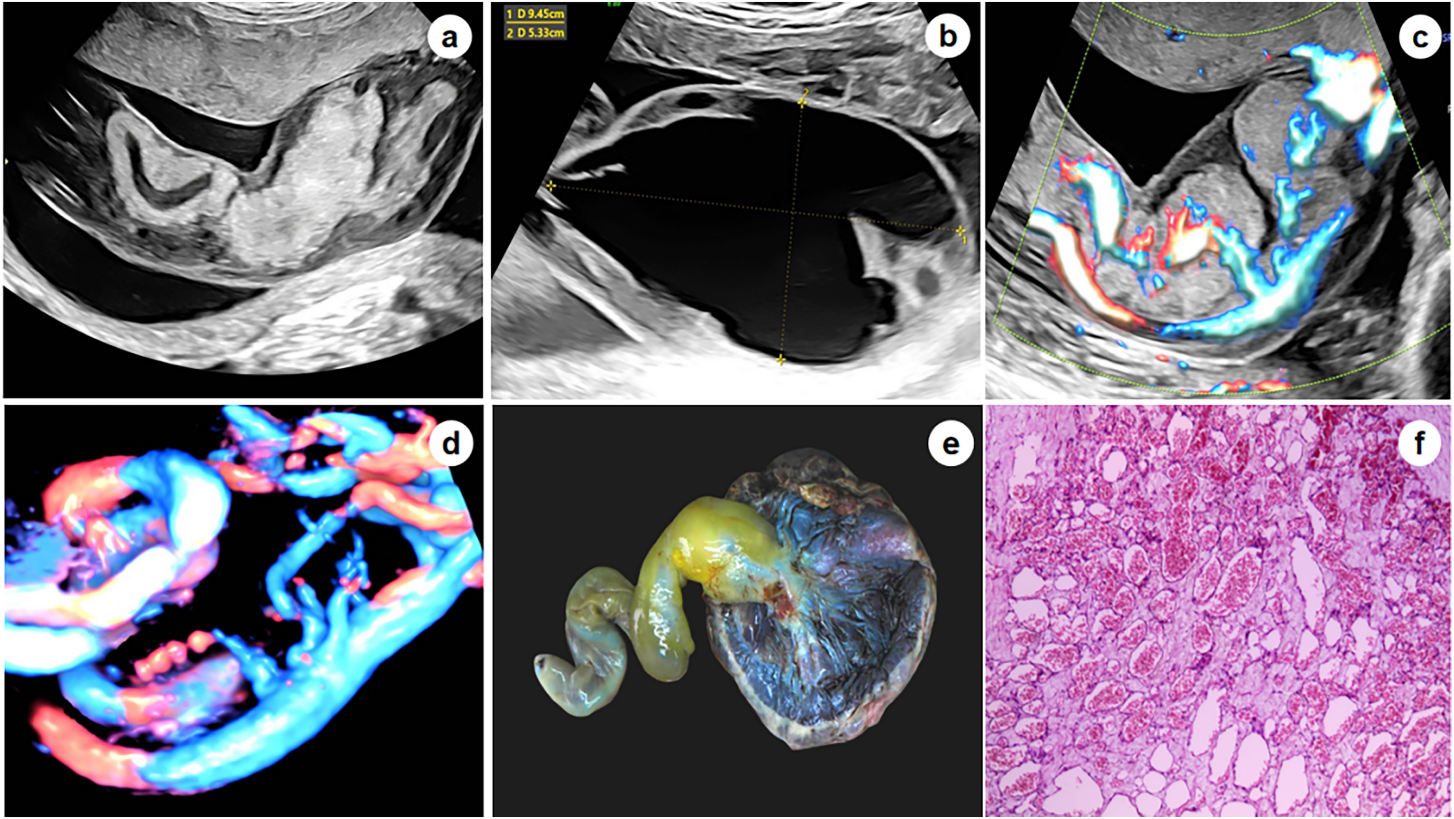
Prenatal ultrasonographic, gross, and histopathological findings of umbilical cord hemangioma in Case 2. Two-dimensional ultrasonography **(a)** showing fusiform enlargement of the umbilical cord, with multiple hyperechoic nodules surrounding the umbilical vessels and associated edema and thickening of Wharton’s jelly. Follow-up ultrasonography **(b)** showing marked enlargement of the cystic lesion. SlowFlow HD imaging **(c)** showing branching intralesional vascular flow originating from the umbilical vessels, and the combination with Spatiotemporal Image Correlation (STIC) **(d)** showing the three-dimensional vascular architecture. Gross examination at delivery **(e)** showing an involved segment extending from the cord root. H&E section (×200) **(f)** showing diffuse proliferation of irregular, thin-walled vascular channels containing erythrocytes within edematous Wharton’s jelly, consistent with umbilical cord hemangioma.

A 35-year-old woman, gravida 2, para 1, underwent a routine ultrasound examination at 19 weeks of gestation, which revealed a markedly thickened umbilical cord extending from the placental insertion site to approximately 6 cm proximal to the fetal umbilicus. The maximal internal diameter measured about 3.2 cm. Within the cord, three hyperechoic nodules were identified encasing the umbilical vessels (shown in Figure 2a), the largest measuring approximately 3.7 × 2.0 cm. Multiple cystic lesions were also observed, while the fetal anatomy appeared normal. Serial ultrasound examinations were performed every 2 weeks. At 29 weeks of gestation, ultrasonography revealed thrombosis of the right umbilical artery and a marked enlargement of the umbilical cord cysts, measuring up to 9.4 × 5.3 cm (shown in Figure 2b). SlowFlow HD imaging demonstrated branching vascular signals arising from the left umbilical artery and vein and extending into the lesion (shown in Figures 2c, d). Fetal biometry was consistent with gestational age, and Doppler assessments of the umbilical artery, middle cerebral artery, and ductus venosus remained within normal limits. Because of the development of right umbilical artery thrombosis and progressive enlargement of the cystic component, follow-up was intensified to weekly ultrasound surveillance. At 32 weeks of gestation, the pulsatility index (PI) of the fetal ductus venosus was elevated to 0.93. The patient was subsequently admitted for close monitoring and received antenatal corticosteroid therapy to promote fetal lung maturation. A live newborn weighing 2,000 g was delivered by cesarean section at 33 weeks of gestation, with Apgar scores of 9 and 10 at 1 and 5 min, respectively.

Pathological examination revealed an umbilical cord with two arteries and one vein (2A1V). A 14-cm segment extending from the placental insertion appeared markedly thickened, with pronounced edema of Wharton’s jelly (shown in Figure 2e). Microscopically, the lesion was identified as an umbilical cord hemangioma with myxoid degeneration (shown in Figure 2f). The tumor showed expansile growth encasing one umbilical artery, whereas thrombotic occlusion with luminal narrowing and fibrin deposition was present in the other umbilical artery. Immunohistochemical staining demonstrated positive expression of CD31 and ERG, supporting the endothelial origin of the lesion. The placenta weighed 302 g (25th–50th percentile for gestational age) and showed delayed maturation, with focal avascular villi, increased intervillous fibrin deposition, and decidual vasculopathy with deficient vascular remodeling. Postnatal examination likewise revealed no cutaneous hemangiomas or other clinically apparent vascular malformations.

## Discussion

Umbilical cord hemangioma is an exceptionally rare benign neoplasm derived from endothelial cells (4). It can occur in any portion of the umbilical cord or, less commonly, extend throughout its entire length (5). Reported lesion sizes vary widely, ranging from 0.2 to 23 cm in diameter (1, 6–9), which may partly reflect the absence of standardized measurement protocols, particularly regarding whether cystic components are included. In our center, both cases exhibited unusually extensive involvement of the umbilical cord.

Despite its benign histologic nature, umbilical cord hemangioma may be associated with significant perinatal morbidity and mortality. A multicenter case series published in 2024 reviewed 56 cases reported over the past 12 years, of which approximately 25% resulted in intrauterine or neonatal death and 13% were associated with severe complications (2). Notably, fetal outcome was not significantly associated with lesion size or anatomical location, suggesting that static morphological features alone are insufficient predictors of prognosis. Common complications included fetal growth restriction, polyhydramnios, umbilical blood flow obstruction, fetal hydrops, preterm delivery, and concomitant vascular malformations (2). Elevated maternal serum alpha-fetoprotein (MS-AFP) levels have also been reported in a substantial proportion of cases (6). In addition, associations with systemic vascular anomalies, such as Klippel–Trenaunay–Weber syndrome and diffuse neonatal hemangiomatosis, have been described (10–12). These findings highlight the importance of comprehensive fetal evaluation when this condition is diagnosed prenatally.

From an imaging perspective, a relatively consistent sonographic pattern can be recognized. Both the literature and our cases demonstrate localized fusiform enlargement of the umbilical cord with mixed echogenicity, including hyperechoic nodular components and cystic changes (13). The hyperechoic areas are typically irregular in shape and often surround the umbilical vessels, while low-velocity intralesional blood flow is commonly detected. Advanced Doppler imaging may reveal fine dendritic vascular branches extending into the lesion and, occasionally, larger neovascular channels within the affected segment. In addition to facilitating visualization of low-velocity intralesional blood flow with HDlive Flow and SlowFlow HD, the incorporation of STIC enables three-dimensional reconstruction of the vascular network and its spatial relationship to the umbilical vessels. This combined approach may improve appreciation of vessel origin, branching patterns, and the overall vascular architecture of the lesion. Akiba et al. reported that HDlive Flow was useful for depicting small vascular branches within an umbilical cord hemangioma when conventional color Doppler imaging was inconclusive (14). Our observations suggest that the addition of STIC may further enhance characterization of complex vascular lesions by providing additional spatial information regarding the extent and organization of intralesional vessels. To place our findings in the context of previous reports, Table 1 summarizes the prenatal ultrasound and Doppler imaging characteristics described in selected cases of umbilical cord hemangioma, highlighting the evolution of imaging techniques from conventional ultrasonography and Doppler studies to advanced three-dimensional vascular imaging approaches. The studies included in Table 1 were identified through a PubMed search of published articles up to June 2025 using the keywords “umbilical cord hemangioma,” “prenatal diagnosis,” and “ultrasound.” Representative reports were selected to illustrate the evolution of prenatal ultrasound and Doppler imaging techniques used for lesion characterization. Studies were included if they provided sufficiently detailed prenatal ultrasound descriptions. Additional Doppler and advanced vascular imaging findings were extracted when reported, allowing comparison of evolving imaging techniques used for lesion characterization. Reports lacking adequate ultrasound information were not included.

**TABLE 1:** Prenatal ultrasound and Doppler imaging findings reported in selected studies of umbilical cord hemangioma.

References	Gestational age at diagnosis (weeks)	Gray-scale ultrasound findings	Doppler and advanced imaging findings	Main imaging contribution
Daniel-Spiegel et al. (13)	28	Heterogeneous fusiform cord mass with a central echogenic component and peripheral multicystic areas	No Doppler findings reported	Demonstrated characteristic prenatal appearance of umbilical cord hemangioma associated with cystic degeneration of Wharton’s jelly
Papadopoulos et al. (6)	22	Hyperechoic mass surrounded by edematous Wharton’s jelly; one umbilical artery traversing the lesion	Serial spectral Doppler assessment demonstrated progressive increase in resistance within the intratumoral umbilical artery	Highlighted the value of targeted Doppler surveillance of vessels involved by the tumor
Smulian et al. (8)	25	Lobulated echogenic mass with solid and cystic components near the fetal cord insertion; associated distal cord edema	Branching arterial vessels with low-resistance flow; three-dimensional power Doppler angiography demonstrated intratumoral vascular branching	Illustrated complex intratumoral vascular architecture using three-dimensional power Doppler imaging
Akiba et al. (14)	19	Hyperechoic lesion associated with umbilical cord cysts	HDlive Flow demonstrated multiple small vessels arising from an umbilical artery and extending into the lesion when conventional color Doppler imaging was inconclusive	Improved visualization of intralesional vascular branches and their relationship to the umbilical vessels
Lok et al. (18)	21	Multiseptate hypoechoic cystic mass containing an echogenic nodule near the fetal cord insertion	Color/power Doppler and three-dimensional color Doppler demonstrated arterial supply, venous drainage, and intratumoral arteriovenous shunting	Facilitated assessment of tumor vascular architecture and vessel involvement
Present Case 1	23	Fusiform enlargement of the umbilical cord with hyperechoic nodules and Wharton’s jelly edema	STIC combined with HDlive Flow demonstrated multiple intralesional vascular channels and their three-dimensional relationship to the umbilical vessels	Provided simultaneous visualization of vascular architecture and spatial vessel relationships
Present Case 2	19	Fusiform cord enlargement with hyperechoic nodules and progressive cystic enlargement	SlowFlow HD demonstrated low-velocity branching vascular flow; STIC enabled three-dimensional reconstruction of the vascular network	Improved depiction of low-velocity intralesional blood flow and overall vascular organization

STIC, spatiotemporal image correlation.

As shown in Table 1, advances in prenatal imaging have progressively improved characterization of the vascular architecture of umbilical cord hemangiomas, from conventional ultrasonography and Doppler studies to three-dimensional Doppler techniques and, more recently, STIC-based vascular reconstruction. Although these advanced imaging modalities may facilitate visualization of low-velocity blood flow and complex vascular anatomy, they should be regarded as adjunctive tools rather than essential components of diagnosis, as most reported cases have been successfully identified using conventional ultrasonography and Doppler imaging alone. Most cases are associated with edema of Wharton’s jelly or pseudocystic changes. Umbilical cord hemangiomas may arise from one or more umbilical vessels and are frequently located near the placental insertion site (8). In our series, one lesion was located near the fetal insertion and the other at the placental end.

The differential diagnosis of umbilical cord hemangioma includes hematoma, umbilical cord cyst, urachal cyst, thrombus, varix, aneurysmal change, teratoma, and metastatic neuroblastoma (1, 15). When the lesion is located near the fetal insertion, careful evaluation is required to exclude omphalocele by assessing abdominal wall integrity and internal echogenic characteristics. Cord pseudocysts usually lack internal vascular signals, whereas umbilical vein varices present as focal venous dilatations without solid nodular components. Hematomas may appear heterogeneous but generally do not demonstrate persistent internal vascular flow. In contrast to these entities, umbilical cord hemangioma typically demonstrates a mixed solid–cystic morphology with internal arborizing vascularity and a close relationship to the umbilical vessels, which may aid in differentiation. In our cases, advanced Doppler imaging combined with STIC provided additional information regarding the intralesional vascular architecture and its spatial relationship to the umbilical vessels. These findings facilitated differentiation from lesions such as pseudocysts, hematomas, and focal vascular dilatations, which generally lack the complex branching vascular architecture observed in hemangiomas. Fetal magnetic resonance imaging (MRI) is not routinely required for the diagnosis of umbilical cord hemangioma, as the lesion can usually be adequately evaluated by ultrasonography and Doppler imaging. However, MRI may be considered in selected cases when sonographic assessment is limited or the extent of the lesion is unclear. In such situations, MRI can provide additional anatomical information and may serve as a complementary tool for prenatal evaluation.

From a clinical and pathophysiological perspective, umbilical cord hemangiomas are frequently associated with pseudocysts and marked edema of Wharton’s jelly, likely due to plasma transudation secondary to persistent vascular perfusion within the lesion (16). Proposed mechanisms of fetal compromise include vascular compression, intravascular tumor proliferation, thrombosis, and vessel rupture (6, 17). As the lesion enlarges, it may compress adjacent umbilical vessels, leading to luminal narrowing or occlusion. Compression of the umbilical vein, in particular, has been associated with a substantially increased risk of fetal demise (4, 18, 19). In Case 1, although no fetal autopsy was performed and alternative causes of intrauterine fetal demise cannot be completely excluded, histopathological findings supported umbilical vessel compression as the most likely mechanism underlying the fetal demise. Placental examination further demonstrated features of chronic fetal circulatory compromise, providing additional evidence of progressive hemodynamic impairment. In Case 2, pathological findings, including umbilical artery thrombosis, focal avascular villi, and decidual vasculopathy, were also consistent with compromised fetoplacental perfusion. Together, these findings suggest that progressive fetoplacental circulatory impairment may contribute to adverse perinatal outcomes in umbilical cord hemangioma. In addition, highly vascular lesions may produce arteriovenous shunting, potentially resulting in fetal cardiac failure and hydrops (16).

Importantly, our first case demonstrated that intrauterine fetal demise may occur despite apparently normal Doppler parameters and the absence of fetal growth restriction. This observation suggests that conventional Doppler indices may not fully reflect evolving hemodynamic compromise in some cases and highlights the need for further investigation.

From a clinical perspective, serial ultrasound surveillance is essential, with particular attention to lesion growth, progression of Wharton’s jelly edema, development of cystic changes, and signs of vascular compromise such as thrombosis or venous compression. Although no validated prognostic markers or standardized management guidelines currently exist for umbilical cord hemangioma, these findings have been associated with adverse perinatal outcomes in reported cases and may indicate an increased risk of fetal compromise. Therefore, rapid lesion enlargement, worsening edema, thrombosis, suspected vessel compression, or abnormal fetal surveillance findings may justify more intensive monitoring, including more frequent ultrasound examinations, Doppler assessment, and fetal heart rate surveillance. Decisions regarding delivery should be individualized, taking into account gestational age, fetal condition, and the balance between the risks of prematurity and continued intrauterine exposure to a potentially progressive lesion. These recommendations are based on clinical experience and limited published evidence.

## Conclusion

Umbilical cord hemangioma is a rare benign vascular tumor with potentially significant clinical consequences. Although histologically benign, adverse outcomes appear to be related primarily to secondary mechanical and hemodynamic disturbances affecting fetoplacental circulation.

Prenatal ultrasound plays an important role in the detection and surveillance of this condition. Recognition of characteristic imaging features, together with careful assessment of vascular changes, may assist in identifying cases at increased risk. Importantly, conventional Doppler parameters may not always reflect evolving vascular compromise, although further evidence is needed to clarify their prognostic value.

Close follow-up focusing on structural progression and signs of vascular compromise may assist clinical management, although evidence-based prognostic markers and standardized management guidelines are currently lacking.

## Data Availability

The original contributions presented in this study are included in this article/supplementary material, further inquiries can be directed to the corresponding author.

## References

[1] Angelico G Spadola S Ieni A Gurrera A Arena MG Arciuolo D et al. Hemangioma of the umbilical cord with associated amnionic inclusion cyst: two uncommon entities occurring simultaneously. *Pathologica*. (2019) 111:86. 10.32074/1591-951X-26-17-EC 31596275 PMC8186010

[2] Maréchal-Ross IC Siva S Mizia K Pulvers JN Turton I Moghimi A. Hemangioma of the umbilical cord: a case report and proposal for standardised reporting criteria. *Case Rep Womens Health*. (2025) 46:e00708. 10.1016/j.crwh.2025.e00708 40256492 PMC12008527

[3] Ferreira EO Stefanovici C Kostadinov S Duncan V. Umbilical cord hemangiomas: a multi-institutional case series with literature review. *Pediatr Dev Pathol*. (2024) 27:569–75. 10.1177/10935266241264161 39056566 PMC11568650

[4] Wanyonyi S Nyagaka F Okiro P Ogutu L Nyaichowa A Oindi F et al. Umbilical cord hemangioma and pseudocyst with favorable fetal outcome. *Clin Case Rep*. (2023) 11:e7656. 10.1002/ccr3.7656 37415590 PMC10319952

[5] Fox H Sebire NJ. Pathology of the umbilical cord. In: Fox H Sebire NJ editors. *Pathology of the Placenta: Major Problems in Pathology.* 3rd ed. London: WB Saunders (2007). p. 473.

[6] Papadopoulos VG Kourea HP Adonakis GL Decavalas GOA. case of umbilical cord hemangioma: Doppler studies and review of the literature. *Eur J Obstet Gynecol Reprod Biol*. (2009) 144:8–14. 10.1016/j.ejogrb.2009.01.011 19251351

[7] Ben Thayer M Helal I Khanchel F Mbarki C Bettaieb H Ben Brahim E et al. Hemangioma of the umbilical cord: a case report on a rare entity. *Clin Case Rep*. (2022) 10:e6441. 10.1002/ccr3.6441 36245456 PMC9547990

[8] Smulian JC Sarno AP Rochon ML Loven VA. The natural history of an umbilical cord hemangioma. *J Clin Ultrasound*. (2016) 44:455–8. 10.1002/jcu.22346 26899634

[9] Lisovaja I Franckevica I Vedmedovska N. Large angiomyxoma of the umbilical cord-uncomplicated rupture of tumor membranes at 32 weeks of gestation. *Diagnostics*. (2022) 12:1339. 10.3390/diagnostics12061339 35741148 PMC9222199

[10] Schwickert A Seeger KH Rancourt RC Henrich W. Prenatally detected umbilical cord tumor as a sign of diffuse neonatal hemangiomatosis. *J Clin Ultrasound*. (2019) 47:366–8. 10.1002/jcu.22689 30673136

[11] Yu D Sun L Chen T. Prenatal ultrasound diagnosis of Klippel-Trenaunay-Weber syndrome associated with umbilical cord hemangioma. *J Clin Ultrasound*. (2021) 49:254–6. 10.1002/jcu.22896 33210306

[12] Sherer DM Al-Haddad S Cheng R Dalloul M. Current perspectives of prenatal sonography of umbilical cord morphology. *Int J Womens Health*. (2021) 13:939–71. 10.2147/IJWH.S278747 34703323 PMC8541738

[13] Daniel-Spiegel E Weiner E Gimburg G Shalev E. The association of umbilical cord hemangioma with fetal vascular birthmarks. *Prenat Diagn*. (2005) 25:300–3. 10.1002/pd.1109 15849800

[14] Akiba Y Miyakoshi K Ochiai D Kawaida M Matsumoto T Tanaka M. Umbilical cord hemangioma: sonographic features by HDlive Flow. *Eur J Obstet Gynecol Reprod Biol*. (2018) 221:195–6. 10.1016/j.ejogrb.2017.12.011 29229175

[15] Iglesias-Deus A Pérez-Muñuzuri A Urisarri A Bautista-Casasnovas A Couce ML. Umbilical cord and visceral hemangiomas diagnosed in the neonatal period: a case report and a review of the literature. *Medicine*. (2016) 95:e5196. 10.1097/MD.0000000000005196 27759656 PMC5079340

[16] Grossi AP Astori AF Nakatani ET Jure R Salazar D Tonni G et al. Prenatal diagnosis of umbilical cord angiomyxoma: case studies and literature review of 45 cases. *J Ultrasound Med*. (2024) 43:1769–84. 10.1002/jum.16506 38884130

[17] Vougiouklakis T Mitselou A Zikopoulos K Dallas P Charalabopoulos K. Ruptured hemangioma of the umbilical cord and intrauterine fetal death, with review data. *Pathol Res Pract*. (2006) 202:537–40. 10.1016/j.prp.2006.02.008 16684589

[18] Lok WY Law KM Ho CL Leung TY. Prenatal diagnosis of umbilical cord hemangioma. *Ultrasound Obstet Gynecol*. (2022) 59:392–3. 10.1002/uog.24755 34435409

[19] Matsuda S Sato Y Marutsuka K Sameshima H Michikata K Ikenoue T et al. Hemangioma of the umbilical cord with pseudocyst. *Fetal Pediatr Pathol*. (2011) 30:16–21. 10.3109/15513811003796920 21204661

